# Gestational monitoring in buffalo females: detection of *Neospora caninum* by PCR

**DOI:** 10.1590/1984-3143-AR2023-0052

**Published:** 2023-11-20

**Authors:** Raúl Miguel Reyes-Sandoval, Dora Romero-Salas, Jenny Jovanna Chaparro-Gutiérrez, Anabel Cruz-Romero, Karla María López-Hernández, Miguel Ángel Lammoglia-Villagómez, Milagros González-Hernández, Marco Antonio Alarcón-Zapata, Rebeca Isabel Vergara-Reyes

**Affiliations:** 1 Laboratorio de Parasitología, Unidad de Diagnóstico, Rancho Torreón del Molino, Facultad de Medicina Veterinaria y Zootecnia, Universidad Veracruzana, Tejería, Veracruz, Mexico; 2 Grupo de Investigación Centro de Investigaciones Básicas y Aplicadas en Veterinaria (CIBAV), Facultad de Ciencias Agrarias, Universidad de Antioquía, Medellín, Colombia; 3 Laboratorio de Seguridad Agroalimentaria, Facultad de Medicina Veterinaria y Zootecnia, Universidad Veracruzana, Veracruz, Veracruz, Mexico; 4 Facultad de Ciencias Biológicas y Agropecuarias, Universidad Veracruzana, Tuxpan, Veracruz, Mexico; 5 Facultad de Agronomía y Veterinaria, Universidad Autónoma de San Luis Potosí, Ejido Palma de la Cruz, San Luis Potosí, Mexico

**Keywords:** Bubalus bubalis, Mexico, abortion, stillborn, bovine buffalo system

## Abstract

This study establishes the serological frequency against *Neospora caninum* on day zero and the presence of *N. caninum* DNA surveyed throughout the gestation of *Bubalus bubalis* females in a bovine buffalo system in the central zone of the state of Veracruz, Mexico. Blood samples were taken from 11 females in 6 different sampling periods and analyzed for *N. caninum* antibodies detection on day zero. DNA detection by PCR was performed on all sampling periods. The gestation months of the females were recorded for five trimesters by ultrasonography, as well as births and pregnancy losses. Recorded seropositivity and positivity for agent DNA were 90.9% (95% CI 58.7-9.7) and 36.3% (95% CI 10.9-69.2), respectively, on day zero. *N. caninum* DNA was detected between 18.1% (95% CI 2.3-51.7) and 45.4% (95% CI 16.7-76.6) over the five trimesters of observation, with three births and three abortions recorded. The studied water buffalo population had a high presence of *N. caninum* antibodies; however, the detection of *N. caninum* DNA remained below 47% in the females. The association was only observed in the detection of DNA with pregnant females (*P* 0.007). Our results support the hypothesis of the resistance of water buffaloes to infection and the onset of clinical signs against infection by *N*. *caninum* even upon a high possibility of infection and reinfection described in this production system in Mexico.

## Introduction

In the worldwide context, the water buffalo (*Bubalus bubalis*) has been considered a booming livestock animal, with an economic importance linked to its nature as a triple-purpose animal for producing milk and meat, in addition to being used as a draft animal (Campanile et al., 2016). Buffalo meat has some advantages over that of other bovines, such as less cholesterol and fat, better processing characteristics, and similar organoleptic properties to beef; additionally, its milk is highly appreciated by its high fat and protein content (Ali et al., 2016). Water buffalo are susceptible to many common bovine infectious diseases, such as bovine neosporosis, considered an emerging disease of worldwide distribution (Dubey, 2003). *Neospora caninum* is a parasitic protozoan from canines of great importance in veterinary medicine for infecting a wide range of warm-blooded animals, mainly cattle. However, it has also been reported in water buffalo and other wildlife, potentially causing clinical manifestations such as abortion (Dubey, 2003). It is generally accepted that the main transmission route of neosporosis is vertical, allowing the parasite to be transmitted from generation to generation and remain indefinitely within herds (Japa et al., 2019). Water buffalo is susceptible to experimental infection by *N. caninum* causing fetal death; vertical transmission seems to occur naturally and episodes of abortions have been reported in *B. bubalis* (Auriemma et al., 2014). It is difficult to establish the presence of *N. caninum* in live animals through traditional methods since the parasites are difficult to isolate, being frequently found in tissues that are not amenable to sampling, such as the CNS (Dubey et al., 2007). By applying ELISA, the detection of anti-*N. caninum* antibodies is considered evidence of exposure to the agent (Almería, 2013); however, low concentration of parasites in tissues along with variations in specificity and sensitivity, neither test can detect infection in fetuses or active infection (Yao et al., 2009). Considering the possibility of using the PCR technique in various tissues and body fluids, combined with the above-mentioned difficulties and problems collecting antemortem tissue samples (Yao et al., 2009), *N. caninum* DNA detection in the blood is a viable option (Okeoma et al., 2004; Reyes-Sandoval et al., 2017).

Ultrasonography (US) is a minimally invasive, precise, and effective technique for early diagnosis of pregnancy that can lower the incidence of abortions induced by rectal palpation. Compared to rectal palpation (35 days), US can diagnose gestation 28 days or even earlier. Most studies addressing the application of transrectal ultrasound for pregnancy diagnosis use cattle; however, more recently, the method has also proved successful and useful in buffalo females (Balhara et al., 2013). Establishing the serological frequency and detecting the protozoan throughout gestation in buffalo females and their calves are key points considering both the *N. caninum* infectivity in water buffaloes and the importance of this livestock. In addition, the potential repercussions should also be analyzed within the buffalo/bovine system in tropical sites, such as the central zone of Veracruz, Mexico.

## Methods

The work protocol was approved by the Bioethics and Animal Welfare Commission of the Faculty of Veterinary Medicine and Zootechnics of the Universidad Veracruzana, following current regulations NOM -062-ZOO-1999 (SAGARPA, 2001).

We carried out cross-sectional and longitudinal study with non-probabilistic convenience sampling in a Livestock Production Unit (LPU) located in the central zone of the state of Veracruz, Mexico. The LPU is in the municipality of Cotaxtla (18°53'20” N 96°09'29” W), at an altitude of 40 MASL, with a dry-regular warm climate, mean temperature of 26°C, and mean annual rainfall of 1,900 mm (INEGI, 2010). The water buffalo are kept in close contact with bovines and canines in an extensive production system. The buffaloes remain in the same paddock for periods between four and six months, without food supplementation, vaccination, or deworming program for neither ectoparasites nor endoparasites.

For serology, blood samples were taken in tubes without anticoagulant by puncture of the jugular veins or central auricular artery. The samples were centrifuged at 1000 g for 10 minutes for serum separation and stored at -20°C.

For PCR, blood samples were taken by the above-mentioned routes in tubes with EDTA anticoagulant through the same spin protocol. The white cell layer was separated by aspiration with micropipettes and sterile tips and stored at -20°C. Sampling for DNA detection of *N. caninum* was performed every three months together with ultrasonography.

An ELISA commercial test (IDEXX®) was used to identify the IgG antibodies against *N. caninum*. The reading was carried out using a Bio-Rad® model 680 spectrophotometer, and the optical density and wavelength were measured through a 650 nm filter.

We applied a PCR-described protocol using the outer primer pairs of the *Nc-5* gene for the first PCR reaction – Np 21 and Np 4 primers, that amplify a 380 bp fragment. The reaction mix contained 12.5 µL of master mix (PCR Master Mix, Promega™), 1 µL of each primer (10 µM), 2.5 µL of nuclease-free water, and 8 µL of blank DNA, with a total volume of 25 µL. The following cycling conditions were considered: 5 min at 95 °C, 1 min at 94 °C, 1 min at 57 °C, 1 min at 72 °C (35 cycles), and 7 min at 72 °C. For the second PCR reaction, we used the Np 9 and Np 10 primers, which amplify a fragment of 224 bp. The reaction mix contained 12.5 µL of master mix (PCR Master Mix, Promega™), 1 µL of each primer (10 µM), 5.5 µL of nuclease-free water, and 5 µL of initial PCR product, with a total volume of 25 µL. The following cycling protocol was applied: 5 min at 94 °C; 30 s at 94 °C, 20 s at 63 °C, 30 s at 72 °C (35 cycles), and 10 min at 72 °C (Reyes-Sandoval et al., 2017). The amplification products were separated on 2% agarose gels stained with ethidium bromide and visualized under ultraviolet light, with a 100 bp molecular weight marker (100bp DNA Ladder, Promega™). *N. caninum* tachyzoites extracted from commercial IFA plates were used as the positive control.

The fieldwork covered from August 2021 to August 2022 by trimesters on five occasions after the serological diagnosis on day zero. The equipment used was an Easi-Scan™ BFC ultrasonography with a 4.5-8.5 MHZ transrectal transducer. Finally, the gestation months, abortions, and births were recorded by ultrasound on each sampling date. The occurrence of an abortion was considered upon no ultrasonographic evidence of gestation in the following sampling dates. Additionally, the calves born within the observation period were sampled before the consumption of colostrum for serological analysis and PCR.

The bands resulting from the PCR test were subjected to automated sequencing (Sanger 2nd generation) and the respective results were compared through BLAST. We performed the sequence alignment on the MEGA 11 program and built a phylogenetic tree from it by comparing our sequence with 8 other sequences from various parts of the world, in addition to different hosts.

The data were analyzed on the SPSS program (Ver. 20.0 IBM Corp.) using the Chi^2^ or Fisher test to define the association between the results of the direct test and the different variables considered.

## Results

**Figure 1:**
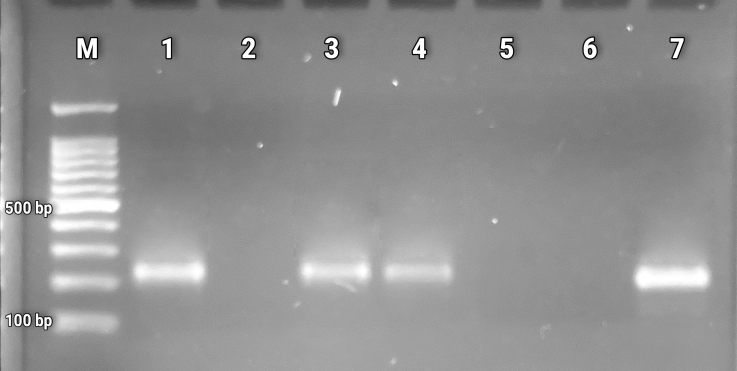
PCR products from leukocytes of naturally infected buffalo females. M molecular marker 100 bp, 1 positive control (*N. caninum* tachyzoites), and 2-7 female samples (date 4).

The tests involved eleven female buffaloes aged from 18 to 135 months kept in the buffalo/bovine system, from which 11 serum samples and 66 white blood cell samples were analyzed. On day zero, the registered seroprevalence and PCR prevalence were 90.9% (95% CI 58.7-99.7) and 36.3% (95% CI 10.9-69.2), respectively. Table 1 shows the PCR test results (Figure 1) and the gestational diagnosis.

**Table 1:** Positivity frequency for PCR and physiological state in buffalo females resulting from gestational follow-up.

**Sampling date**	**“n”**	**Positives**	**Frequency %**	**CI 95%**	**Pregnant**	**Abortion**	**Birth**
**1**	11	3	27.2	6.02-60.9	3	0	0
**2**	11	5	45.4	16.7-76.6	3	1	0
**3**	11	3	27.2	6.02-60.9	8	1	2
**4**	11	3	27.2	6.02-60.9	9	0	0
**5**	11	2	18.1	2.3-51.7	8	1	1

CI: Confidence Interval 95%

Three births and three abortions were registered during the observation period. One birth was a stillborn calf and the other two were healthy. Although all calves showed *N. caninum* antibodies, *N. caninum* DNA in blood was only found in two calves, the stillborn and one healthy.

In turn, based on the ultrasound findings in follow-up assessments, three abortions were recorded, one during the first pregnancy trimester and the other two in the second trimester. Since the animals live in an extensive grazing system and the abortions were neither observed nor reported by LPU workers, the aborted fetuses could not be recovered for further analysis. Even though the three females that suffered abortion had antibodies at the beginning of the study, *N. caninum* DNA was found in the blood of only two of them.

The Chi^2^ test showed an association between the results of *N. caninum* DNA detection by PCR and the reproductive status, that is, pregnant females (Chi^2^ 14.15; *P* 0.007). In contrast, no association of the presence of *N. caninum* DNA was found with the sampling date (Chi^2^ 0.91; *P*0.92) or with the birth of offspring; although, in this case the Fisher test was considered by the number of frequencies (*P* 0.41).

**Figure 2:**
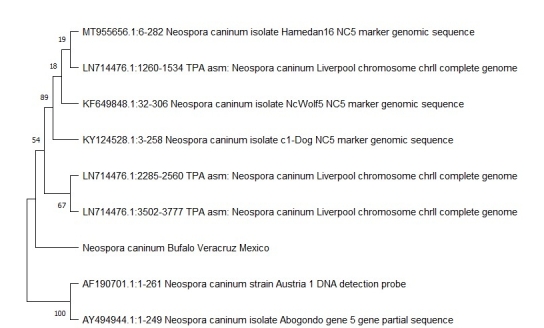
Phylogenetic tree of *N. caninum* based on repetitive the *Nc5* gene. Values on the tree nodes correspond to bootstrap proportions (%). Identified isolates characterized by geometric shapes. The different isolates are shown with their accession number in the GenBank.

The Blast tool returned a similarity ranging from 90 to 93% (accession numbers: MG973172.1; MT709296.1; KX683873.1; KF649847.1; KU253799.1; MT709298.1; KF649848.1; MT955656.1), with the highest value for the sequence described in a work from Iran (Figure 2).

## Discussion

To the authors' knowledge, this is the first report on the detection of *N. caninum* DNA throughout gestation in naturally infected buffalo cattle and their offspring.

There is a great variation in the seroprevalences described in water buffalo, ranging from 0% to 88.3% (Reichel et al., 2015; Neverauskas et al., 2015). Our findings pointing to 90.9% seropositivity exceed the highest reported seroprevalence, which could be explained by some differences in the studies. For example, the animals in our study aged up to 135 months. It is worth noting that the possibility of presenting antibodies against *N. caninum* increases with age (Dubey, 2003; Dubey et al., 2007; Almería, 2013; Moore et al., 2014; Reichel et al., 2015).

Worldwide, it has been reported that buffaloes have a greater possibility of presenting anti-*N. caninum* antibodies, 3 to 7 times more than cattle (Reichel et al., 2015), and seropositivity in water buffalo has been estimated to be much higher than that reported in cattle when both species cohabit (Moore et al., 2014). This postulate has been recently ratified in a study carried out in Mexico, which reported high seropositivity when the two bovine species coexist closely (Reyes-Sandoval et al., 2021), thus corroborating our study.

Internationally there’s has been highly variable reports on anti-*Neospora* antibodies in cattle, for instance from 2.8% in Nigeria’s autochthonous breeds (Ayinmode and Akanbi, 2013) to 28.6% or 37% in South America [Moore et al. (2014) and Chaparro et al. (2017) respectively]. In general, this variation is depending on the region, the type of cattle involved, and the diagnostic test used. It should be noted that studies that consider the production systems where buffaloes and cattle coexist and especially in the case of tropical, extensive or dual-purpose livestock are minimal.

Since it is believed that the situation is less important in beef cattle compared to dairy cattle kept in intensive production few studies have focused on *N. caninum* and neosporosis in tropical systems in Mexico. In such tropical systems in southeast Mexico, a general prevalence of 11.6% has been reported (García-Vázquez et al., 2009) but prevalence’s varies depending on the state were performed from 8.6% (Veracruz), 15% (Chiapas) to 11.3% [Yucatan; García-Vázquez et al. (2009)] even in the same state prevalence reports varies, for example from 8.6% to 26% [Veracruz, García-Vázquez et al. (2009) and Romero-Salas et al. (2010) respectively].

Other studies carried out in Veracruz state recorded *N. caninum* prevalence of 20.8% in dual-purpose cattle (Montiel-Peña et al., 2011) and 26% in dairy cattle, beef cattle and their crosses with the highest percentage of seropositivity recorded in five-year-old females and the lowest in one-year-old females (Romero-Salas et al., 2010).

Additionally, in more recent studies reports 29.4% *N. caninum*-positive in dual-purpose cattle and water buffalo ranch where animals shared habitat (Reyes-Sandoval et al., 2021); the seroprevalence against *N. caninum* in herds where buffaloes and cattle are living together is greater than when each species is kept separately, but not so with dairy cattle, either on a small scale or intensive system (Salguero-Romero et al., 2021).

The high percentage of antibodies in buffalo females could be explained by the high possibility of constant reinfection due to the consumption of water or food contaminated with oocysts. The animals included in our study graze on induced meadows and with native grasses; in addition, the habits of the buffalo species are kept near or within dams that may also be contaminated by the mechanical dragging of oocysts through the water (Neverauskas et al., 2015).

As to the detection of *N. caninum* DNA and respective antibodies, our findings for the specimens used in this study reached 36.6% and 90.9%, respectively. Such results vary greatly from some previous reports on dairy cattle [e.g., 89% and 85%, respectively, in state of Mexico; Reyes-Sandoval et al. (2017)] but are close to some others [e.g., 35% and 100%, respectively, in Aguascalientes; Santana et al. (2010)]. However, *N. caninum* is known for being easily transmitted vertically and remaining up to 100% of replacement heifers in intensive dairy systems (Dubey, 2003; Japa et al., 2019).

The low detection of DNA has been related to the production system because the contact with sporulated oocysts of *N. caninum* is much more possible in extensive or small-scale dairy systems where animals can graze on contaminated grasslands or water (Dubey et al., 2007) than technological or intensive systems (Reyes-Sandoval et al., 2021). Another important factor is the age of the females, within the present study there are females with more than 10 years. In most of the serological and direct diagnostic studies, it has been established that the possibility of presenting antibodies and/or detecting *N. caninum* DNA will increase with age and the number of births, a situation that could be verified in dairy cattle and buffalo (Dubey, 2003; Dubey et al., 2007; Almería, 2013; Moore et al., 2014; Reichel et al., 2015).

As to a low detection of DNA in female *B. bubalis*, despite a higher seroprevalence, water buffalo has been indicated to be more resistant to the reproductive consequences caused by *N. caninum* infection, with lower abortion rates and higher calving and weaning rates than cohabiting domestic cattle. Hence, *B. bubalis* is suggested to be resistant to *N. caninum* (Moore et al., 2014; Reichel et al., 2015). Vertical transmission and the occurrence of abortions linked to *N. caninum* have been demonstrated even considering the species’ feeding and resting habits (Guarino et al., 2000; Nasir et al., 2011; Reichel et al., 2015).

Concerning the intermittent detection of agent DNA, it worth considering that given the host's high immune response, the protozoan could pass into its resistance form or bradyzoite and become encysted in various tissues, so that later, upon low activity of the immune system, it reactivates the infection (Dubey, 2003; Dubey et al., 2007; Reichel et al., 2015).

The positive results of serology and PCR in the three calves born reiterate the *N. caninum*’s high capacity for vertical transmission. The presence of circulating antibodies before the intake of colostrum, in the case of healthy calves, indicates that infection occurred in the third gestation trimester, reflecting the maturity of the immune system of the fetuses. In addition, the mother’s immune system controls the infection and avoid abortion (Dubey et al., 2007). Furthermore, the virulence of the *N. caninum* isolate might have been low and able to generate infection without the death of the fetus and subsequent abortion. Thereby, variations have been described in the morphological, biological, and pathogenic characteristics of different isolates, which are also influenced by the fact that the isolates come from animals that either show clinical signs of infection or not. *In vitro* or *in vivo* studies of *N. caninum* pathogenicity using animal models generally suggest a deficiency in the capacity to generate the infection and develop the disease. Additionally, it is worth considering that each host is differently susceptible depending on age, breed, zootechnical purpose, immune status, among others (Al-Qassab et al., 2010). In cattle, many studies have focused in assessing vertical transmission, with varying results, such as those reported for the pathogenicity of *N. caninum*. Reports have indicated offspring with anti-*N. caninum* antibodies from seropositive and seronegative mothers; in contrast, animals without antibodies from seropositive mothers have also been reported. In addition, both high and low concentrations of antibodies have been described in all cases (Macedo et al., 2013).

The detection of *N. caninum* DNA shows a similar scenario either in blood or tissues through different PCR protocols (Macedo et al., 2013). A lack of relationship between mothers’ serology and PCR results in their fetuses has been indicated, as a high percentage of infected fetuses came from serologically negative mothers (McInnes et al., 2006). Herein, all three calves came from seropositive mothers and also presented antibodies; however, DNA in circulating blood was detected in only two of them. A negative PCR result in blood samples does not exclude infection by *N. caninum* since it would imply a low proportion of circulating tachyzoites, hence a difficulty in diagnosis, even using molecular techniques or a variable concentration of *N. caninum* DNA in blood samples (Yao et al., 2009).

The results of serology and PCR for the stillborn calf were equal to those for the healthy calves, thus suggesting the potential occurrence of the same infection. However, many possible agents can cause stillbirth. Since the buffaloes used in this study had no food supplementation, no antiparasitic treatment, and no vaccination program, there is a great possibility of bacterial and viral infections, besides *N. caninum*, food deficiencies, among others (Dubey, 2003; Dubey et al., 2007). Our study could only demonstrate the presence of antibodies and parasitemia in the stillborn calf, confirming vertical transmission in a naturally infected water buffalo.

Abortions have been recorded during the three gestation trimesters in bovines and water buffalo in all production systems (Dubey et al., 2007; Auriemma et al., 2014; Campanile et al., 2016). The three pregnancy losses described here fit the descriptions and characteristics of time of occurrence in buffalo. Despite the apparent resistance of *B. bubalis* to neosporosis, *N. caninum* has shown to cause abortion (Guarino et al., 2000; Nasir et al., 2011). There is a great possibility that *N. caninum* is involved in these pregnancy losses since more than 90% of the females presented antibodies, which has been associated with abortion (Dubey et al., 2007; Reichel et al., 2015), along with the intermittent detection of DNA throughout the observational period in all the females, which, in turn, has also been described in gestational follow-ups (Santana et al.*,* 2010; Reyes-Sandoval et al., 2017).

A comparison of the *Nc5* gene sequence obtained herein with partial and complete genomes pointed to the closest proximity with European isolates (accession number AY494944.1) and the furthest with Iranian isolates (accession number MT955656.1). It is worth noting that the sequences shown in Figure 2 originate both from different definitive hosts, such as wolves, coyotes, and dogs, and intermediate hosts, such as bovines and buffaloes. Such results demonstrate the diversity of *N. caninum*.

*N. caninum* has shown a great genetic diversity for different isolates and respective origins, indicating that its ability to infect and develop the disease in such a wide variety of definitive and intermediate hosts must be related to its genetic diversity (Al-Qassab et al., 2010; Calarco and Ellis, 2020). A comparison between the alignment of the nucleotide sequence by PCR and the sequences deposited in GenBank (NCBI) showed a similarity ranging from 90% to 93%. Similar variations have been reported in some studies using the repetitive *Nc5* gene, whereas minor differences between *N. caninum* isolates ranging from 95–100% have been detected. Between 95 and 99% were derived from fox and coyote oocyst DNA, revealing a variation of only 6 bp in five NC1 isolates from Brazilian buffaloes (Al Qassab et al., 2010; Salehi et al., 2021). The phylogenetic analysis shows that the closest isolate belongs to Iran (Figure 2), notably not far from other isolates originating from different species, such as dogs, coyotes, wolves, and cattle. Undoubtedly, this type of study should be expanded in Mexico to learn more about the genetic features and diversity present in the different areas and species linked to livestock.

Our results suggest that both horizontal and vertical transmission occur in *B. bubalis* kept in the buffalo/cattle system present in a tropical site in central Veracruz, Mexico. The population of water buffaloes show a high presence of antibodies; however, the detection of *N. caninum* DNA remained below 46% in the studied females. Such a finding supports the hypothesis of the resistance of water buffaloes to the onset of clinical signs against infection by *N. caninum*, even upon a high possibility of infection and reinfection in this production system in Mexico. Finally, there is a marked difference between the DNA sequences found here and those described for other countries.

## Conclusion

Our results suggest that *N. caninum* horizontal and vertical transmission occur in *B. bubalis* kept in the buffalo/cattle system present in a tropical site in central Veracruz, Mexico. The vertical transmission had negative reproductive effects, as abortion and stillborn, in female buffaloes. Furthermore, based on the tropical extensive grazing system with potential intermediate hosts (cattle, buffalo, or wildlife) and wild or domestic canids interacting to maintain the parasite’s life cycle, the epidemiological state of *N. caninum* may be worse than previously reported, however, more research will be necessary to confirm this in the future.
